# Indoor simulations reveal differences among plant species in capturing particulate matter

**DOI:** 10.1371/journal.pone.0177539

**Published:** 2017-05-16

**Authors:** Jungang Chen, Xinxiao Yu, Huaxing Bi, Yanlin Fu

**Affiliations:** 1 College of Soil and Water Conservation, Beijing Laboratory of Urban and Rural Ecological Environment, Beijing Forestry University, Haidian District, Beijing,China; 2 Beijing collaborative innovation center for eco-environmental improvement with forestry and fruit trees, Beijing, China; 3 College of Forestry, Beijing Forestry University, Haidian District, Beijing, China; University of Tennessee Health Science Center, UNITED STATES

## Abstract

A number of studies have focused on the capacity of urban trees and shrubs to serve as efficient biological filters to mitigate air pollution. In this study, five different tree species were assessed for this function. *Kerria japonica*, *Sophora japonica*, *Philadelphus pekinensis*, *Gleditsia sinensis*, and *Prunus persica* '*Atropurpurea*' were tested in a deposition chamber using (NH_4_)_2_SO_4_ particles. We quantified and compared the capability of all tested trees to remove particles by assessing deposition velocity, a measure of the ability to remove particles. When placed in the deposition chamber, *S*. *japonica* had the greatest deposition velocity, followed by *Philadelphus pekinensis*, *G*. *sinensis*, *Prunus persica* '*Atropurpurea*,' and *K*. *japonica*, in descending order. In addition, the comparison of deposition velocities among these species suggested that certain leaf geometries and surface characteristics of broadleaf trees, such as trichomes and grooves, increased particle capture. However, these results change under a different simulation condition using ambient air, suggesting that some trees actually increase pollutant number concentrations more than reduce particle concentration. This outcome can be explained by the aerodynamic effect of trees exceeding the filtering capacity of vegetation under some conditions. This highlights the difficulty of generalizing species selection criteria for practice use. Accordingly, our results indicate that using vegetation to reduce particle pollution and improve the air quality is not a universally advisable and viable solution.

## Introduction

Since 2012, many cities in China have been experiencing severe and persistent haze pollution events, affecting 1.3 million km^2^ and 800 million people [1]. Ash haze appears when air pollution intensifies, seriously affecting people’s lives and presenting elevated health concerns. Acute pollution leads to many adverse physical reactions and diseases. For example, global incidences of alveolar inflammation, respiratory-tract infections, and acute cardiovascular disorders have significantly increased over the past few decades [2–5]. As society has become more aware of the seriousness of these health risks, a number of measures have been taken to mitigate particulate air pollution. These include energy efficiency improvements, emission reductions, cleaner energy sources, sustainable production, and biological control measures [6]. The ability of vegetation to capture atmospheric particulate matter (PM) has also attracted attention [7–9].

Many countries have plans to begin afforestation or tree-planting in order to reduce air particle pollution. Vegetation can serve as a sink for atmospheric PM and is an interface that can absorb organic matter, chemicals, and heavy metals that adhere to PM [10]. The large total leaf area, branches, and complex structures of trees capture air particles [11–13]. This occurs mainly as turbulent air movements occur within the forest canopy and increase PM deposition on leaves. Thus, plants effectively capture PM from the air, thereby improving urban environmental quality.

The leaf, bark, and branch surfaces of trees and shrubs accumulate PM through dry deposition. Many studies have examined differences among plant species in the accumulation of PM on leaves. For example, Sæbø et al. [14] examined PM accumulation by 47 woody species and found that PM accumulation by conifers was greater than that by broadleaf species. Tallis et al. [10] and Dzierżanowski et al. [15] also ranked conifers highest in accumulated PM on foliage. This may be because long, narrow needles, even though they lack hairs or rough surfaces, are more easily struck by airborne PM than are larger and flatter leaves with thicker boundary layers. Canopy area, species composition, forest porosity, and understory vegetation structure and composition are important factors influencing PM interception effects at the forest-stand level [16, 17]. For individual trees, foliage characteristics strongly influence surface particle deposition [18–20]. Leaves with hairs act as filter screens, increasing the deposition of PM on leaf surfaces compared to flat surfaces [21, 22]. The overall anatomical structure of leaves can markedly affect particle interception by plants.

Considerable field sampling research has also been conducted. In this type of research, leaves were sampled from trees grown in some specific regions or urban areas, and then filtering and weighing methods were used to examine the distribution of particles or chemicals deposited on the tree leaves in order to assess the ability of various plant species to accumulate PM [14, 15]. There are several benefits of using sampled leaves to infer particle sources and size distributions in the areas where the sampled leaves grew. This approach permits comparisons of the spread of particles and chemical element compositions among different sampling regions, but this is an inappropriate method for measuring the capacity of plant species to remove particles. Accordingly, some wind tunnel experiments have used deposition velocity and capture efficiency to quantify the relative effectiveness of contrasting species in particle uptake [11, 16–20]. In these studies, wind tunnels are powerful tools for studying fluid dynamics of particles through trees and for investigating particle deposition on trees exposed to omni-directional wind and particles.

The main objective of the present study was to quantitatively assess the capability of trees to remove particles of different sizes. A deposition chamber was specially designed to test the particle deposition velocities of five tree species. Previous research has revealed deposition onto the surfaces that surrounded trees or groups of trees (i.e., the wind tunnel floor, walls, and ceiling) [17, 20]. Accordingly, we have modified the standard method for calculating deposition velocity (*V*_d_), and experimental conditions were improved to account for the various empirical difficulties (e.g., wall loss) in our previous studies. Furthermore, the influence of several anatomical leaf characteristics on *V*_d_ were studied. Five broadleaved species, *Kerria japonica*, *Sophora japonica*, *Philadelphus pekinensis*, *Gleditsia sinensis*, and *Prunus persica* '*Atropurpurea*', were selected as test species owing to their prevalence in north China.

## Materials and methods

### Ethics statement

This study, which was conducted at and approved by Beijing Forestry University, did not involve any endangered or protected species.

### Plant material

Four-year-old saplings of *Kerria japonica*, *Sophora japonica*, *Philadelphus pekinensis*, *Gleditsia sinensis*, and *Prunus persica* '*Atropurpurea*' were used as test specimens in this study. These plants were carefully excavated from the Beijing Forestry University forestry farm in Jiufeng National Forest Park (E 116°28′, N 39°54′) and transported to Beijing Forestry University. The simulation experiments were conducted in the following way. First, all the plants were placed outdoors, with regular watering. Second, after three weeks indoors, it was confirmed that the outdoor plants had adapted to their environmental conditions and that their growth was satisfactory. All the saplings were repotted after they had adapted to the outdoor ambient environment and were then individually replanted into and grown in 21-L plastic pots (30 cm in height and 30 cm in diameter) filled with raw field soil. All the saplings were then moved inside the greenhouse and watered to prevent drought. Saplings were maintained for four to six weeks until being moved to growth chambers, where they were cultivated for one week before the experiment started.

The growth chambers were adjusted to a standard relative humidity of 50%. Lights were turned off from 23:00–02:00, after which the illumination level started to rise until 06:00, when it reached a maximum PAR level of 365 μmol·m^-2^·s^-1^. This illumination level was maintained until 18:00 and then decreased gradually until reaching complete darkness at 23:00. The chamber temperature was 14°C from 00:00 to 01:00, decreased to 11°C from 00:00 to 04:00, and then linearly increased to a maximum level of 25°C by 10:00. The temperatures started to decrease linearly towards 19°C at 18:00. Light and temperature conditions simulated typical conditions of May in Beijing.

### Overall description of the experimental setup

**Fig 1:**
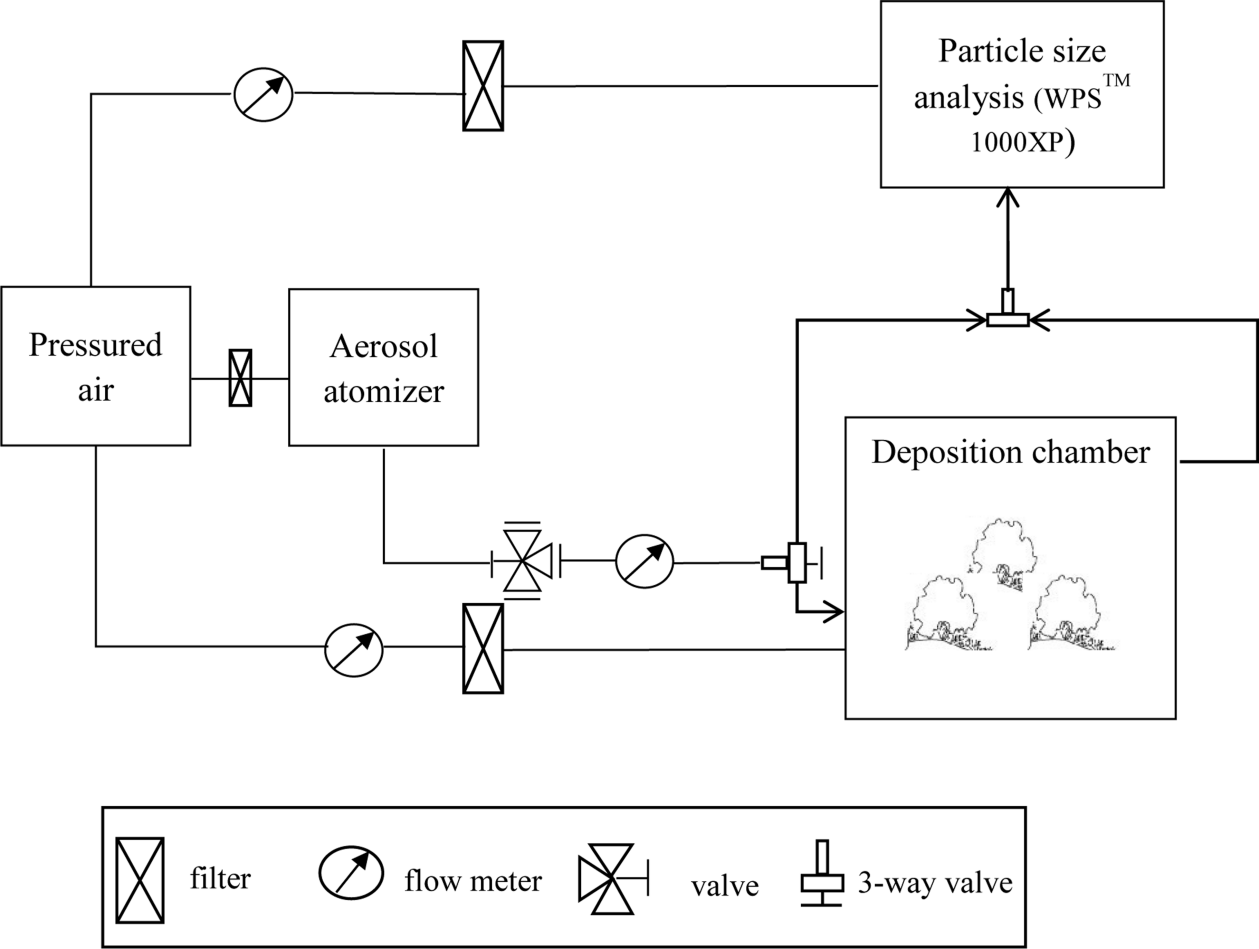
Schematic of the experimental setup.

Experiments were carried out in a deposition simulation chamber facility at Beijing Forestry University. The deposition chamber was designed to simulate omnidirectional air flow around the trees, and the experimental setup is shown in Fig 1. Five saplings for each of the five tree species were individually exposed to dry (NH_4_)_2_SO_4_ aerosol particles with a 10-nm to 10-μm mean diameter range in the deposition chamber (1 m × 1 m × 1 m, FEP Teflon, DuPont, Wilmington, DE, USA). Stainless-steel circular tubes were used as the inlet and outlet of the deposition chamber, placed at the lower and upper parts of each side of the chamber. The inlet and outlet tubes were positioned in such a way as to produce omnidirectional flow in the deposition chamber.

The aerosol particle range was produced by two polydisperse aerosol atomizers, a Six-Jet Atomizer 9306 and a Large Particle Aerosol Atomizer 8108 (TSI, Shoreview, MN, USA). The Six-Jet Atomizer 9306 is used to generate a polydisperse aerosol in high concentrations, producing particle concentrations greater than 106 particles/cm^3^ with a particle size range of 0.01–2 μm. The Large Particle Aerosol Generator 8108 produces a highly concentrated aerosol with a broad particle-size range, from 0.1 to 10 μm in diameter. Before use, the air in the inlet of the aerosol atomizers was purified by an adsorption dryer (KEA 70; Zander Aufbereitungstechnik GmbH & Co. KG, Essen, Germany). The aerosol through the sampling inlet was drawn into a mixing chamber by a fan. The polydisperse aerosol was classified by the DMA according to electric mobility of particles and diluted with clean air. This aerosol was measured by a condensation particle counter upstream of the deposition chamber for 5 min and averaged. The flowrates of the polydisperse aerosol mixtures were 2, 4, and 12 L·min^-1^, achieving particle number concentrations of the diluted aerosol flow of about 4100 cm^-3^, 3000 cm^-3^, and 800 cm^-3^, respectively. After 10 min elapsed for stabilization, the particle number concentration downstream of the deposition chamber was measured for 3 min and averaged.

Additional air streams were conducted through a filter (Sol-Vent DCF; Gelman Sciences, Ann Arbor, MI, USA) and introduced into the two simulation chambers. One airstream introduced CO_2_, while the second controlled the relative humidity (RH). The filter was used to remove background particles from the air before entering the chamber. The air flow used for humidification (3.5 L·min^-1^) was controlled by mass flow controllers. By regulating the water vapor, the photosynthetic photon flux density (PPFD)-dependent transpiration of the plants was appropriately compensated for and RH was maintained at constant levels of around 60%. The CO_2_ concentration in the two chambers was kept at levels of about 350 ppm. Halogen lamps (100 W; Yaming, Shanghai, China) were setup outside of the deposition chambers and used to simulate the solar light spectrum though the FEP Teflon roof in order to maintain optimal photosynthesis. At full illumination and at typical mid-canopy heights, the PPFD was 450 μmol·m^-2^·s^-1^ inside the 1000-L chamber.

### Analysis of water-soluble inorganic ions

In order to investigate the total particle deposition on leaf surfaces, we quantitatively analyzed the composition of the aerosol (NH_4_)_2_SO_4_ aerosol particles. The water-soluble inorganic NH_4_^+^ and SO_4_^2-^ ions were used to determine the composition.

Leaves were placed into clean beakers, washed with 20 mL of deionized water, ultrasonically extracted for 10 min, and mechanically oscillated for 20 min. Then the solution in the breaker was transferred into a 20-mL PET vial. The samples were then stored in a freezer at -20°C until ionic analysis. A syringe was used to collect a sample of the washing water, which was run through a Type 0.22-μm filter and extruded into a sample tube. The PTFE filters were used to remove the insoluble particles and filter chips in the extraction prior to analysis by ion chromatography (DX-120, Dionex, Sunnyvale, CA, USA). NH_4_^+^ and SO_4_^2-^ were analyzed; anions were detected with an IonPac-ASll (4 × 250 mm) separation column (Dionex), while cations were detected with an IonPac-CS12A (4 × 250 mm) separation column. The detection limits for NH_4_^+^ and SO_4_^2-^ were 0.01–1000 g/m^3^, and the correlation coefficients between the standard curves exceeded 99.9%. To decrease the measuring error further, four continuous repeated measures of each sample were conducted, thereby achieving a relative standard deviation of the measured insoluble inorganic ions of 0.35–1.41%.

### Calculation of particle deposition velocity

Plants often have high particle capture efficiencies, but few studies have examined the particle capture rates. In our study, we used an empirical formula to estimate the deposition velocity of tree species and to infer differences among tree species. Leaves were scanned and projected area (*A*_p_) was determined with a scanner (HP Scanjet 4850, China Hewlett-Packard Co., Ltd., Beijing, China); then the leaf surface areas were calculated using Photoshop CS6 software (Adobe, San Jose, CA, USA). As all tested saplings were broadleaf species, broadleaf total area (*A*_t_) was calculated by multiplying *A*_p_ by two, corresponding to the upper and lower blade surfaces of each leaf.

In order to evaluate particle loss in the sampling and deposition chamber, the collection efficiency of the empty deposition chamber, *χ*_e_, was estimated using the following formula for all tested particle sizes and flowrates,

$$\chi_{e}=\frac{C_{in,empty}-C_{out,empty}}{C_{in,empty}}$$ (1)

where, *C*_in,empty_ and *C*_out,empty_ are the upstream and downstream particle number concentrations, respectively.

Generally, deposition velocity can describe the particle deposited on the underlying surface and the degree of particle deposition on a surface, when the surface is exposed to an aerosol flow. In other words, deposition velocity can represent the number of particles deposited per unit surface area and per unit time. Therefore, the ability of tree leaves to remove particles was estimated quantitatively in terms of deposition velocity (*V*_d_), which is defined as follows [21, 22],

$$V_{d}=\frac{N_{de,leaves}}{A_{de,leaves}C_{u}t}$$ (2)

where *N*_de,leaves_ is the number of particles deposited on tree leaves, *A*_de,leaves_ is the total leaf surface area, *C*_u_ is the particle number concentration deposited on the leaf surface, and *t* is the time over which particle deposition occurred.

The number of particles injected into the deposition chamber, i.e., *N*_in leaves_, was calculated as

$$N_{in,leaves}=C_{in,leaves}Q_{a}t$$ (3)

where *C*_in,leaves_ is the particle concentration measured upstream of the deposition chamber and *Q*_a_ is the aerosol flowrate of the deposition chamber. The particle number exiting the deposition chamber, i.e. *N*_out,leaves_, was obtained as

$$N_{out,leaves}=C_{out,leaves}Q_{a}t$$ (4)

where *C*_out,leaves_ is the number concentration of particles measured downstream of the deposition chamber. The aforementioned formulas assume that all the particles that did not exit the chamber were deposited on the leaf surface, but that is inaccurate; the particles injected into the deposition chamber were deposited not only on the tree leaves but also the walls of the deposition chamber and sampling tubes. In order to more accurately compare the influence of trees tested in the chamber, the number of particles deposited only on the tree leaves, i.e., *N*_de,leaves_ was estimated as follows by considering *χ*_e_,

$$N_{de,leaves}=N_{in,leaves}(1-\chi_{e})-N_{out,leaves}=[C_{in,leaves}(1-\chi_{e})-C_{out,leaves}]∙Q_{a}t.$$ (5)

It was assumed that particle number concentration around leaves was equal to the particle number concentration upstream of the deposition chamber, namely, *C*_u_ = *C*_in,leaves_. The deposition velocity (*V*_d_) can then be calculated by plugging Eq (5) into Eq (2) as follows

$$V_{d}=\frac{Q_{a}}{A_{de,leaves}C_{in,leaves}}[C_{in,leaves}(1-\chi_{e})-C_{out,leaves}].$$ (6)

### Analysis of leaf surface characteristics

To test the relationship between leaf microstructure and particle accumulation, samples from each of the five species were imaged using a scanning electron microscope (S-3400NII, Hitachi Japan Co., Ltd., Tokyo, Japan). Appropriate leaves were picked and immediately placed into a plastic automatic sealing bag to protect them and minimize the destruction of leaf hairs. Samples were then cut from fresh leaves on both sides of the midrib; each piece was approximately 5-mm square. Each square was then fixed in a 2%-glutaraldehyde solution, rinsed with a phosphoric acid buffer solution (PBS) three times, and dehydrated using a series of ethanol solutions across a gradient of four concentrations: 70%, 80%, 90%, 95%, and 100%. The samples were then sputter coated with metal, and the scanning electron microscope was used to image the leaf surface and select the appropriate ratio for photographs.

### Comparative trial

All above experiments were only conducted under a certain set of fluid mechanics parameters, and the underlying conclusion of the experiments may not be appropriate across all environments (i.e., ambient air). Accordingly, we also conducted a comparative test in the same indoor chamber to determine whether plants can mitigate atmospheric particles and improve air quality. In these trials, outdoor ambient air (about 20 L·min^-1^) was pumped through 10-mm diameter Teflon tubes into the chamber. To compare this with the interception effect of plants, a blank chamber was used at the same time. Particle absorption was observed by measuring aerosol particle number size distributions using a 1000XP Wide-range Particle Spectrometer (MSP Corporation, Shoreview, MN, USA).

Outdoor air was continually pumped into the chamber, and each set of plants was continuously maintained in the chamber from approximately 8:00 to 20:00. By comparing the number size distribution of the chambers with a plant and the chamber without a plant, we can determine whether trees are able to clean air by filtering out pollutants.

The simulation chambers were adjusted to maintain a standard relative humidity of 50%. Lights were turned off from 23:00 to 02:00, after which the illumination level began to rise until 06:00, when it reached a maximum PAR level of 365 μmol·m^-2^·s^-1^. This level was maintained until 18:00 and then decreased gradually until reaching total darkness at 23:00. The chamber temperature was 14°C from 00:00 to 01:00, decreased to 11°C from 00:00 to 04:00, and then linearly increased to a maximum temperature of 25°C by 10:00. Temperatures began to decrease linearly towards 19°C at 18:00. Light and temperature conditions simulated typical conditions for May in Beijing.

### Statistical analysis

Statistical analyses were conducted with SPSS PASW 18.0 (SPSS Inc., Chicago, IL, USA). One-way analysis of variance (ANOVA) was used to test for significant differences among the five plant species in retention of size-fractionated particles.

## Results

### Variation in particle number concentration and mass deposited on leaves

**Fig 2:**
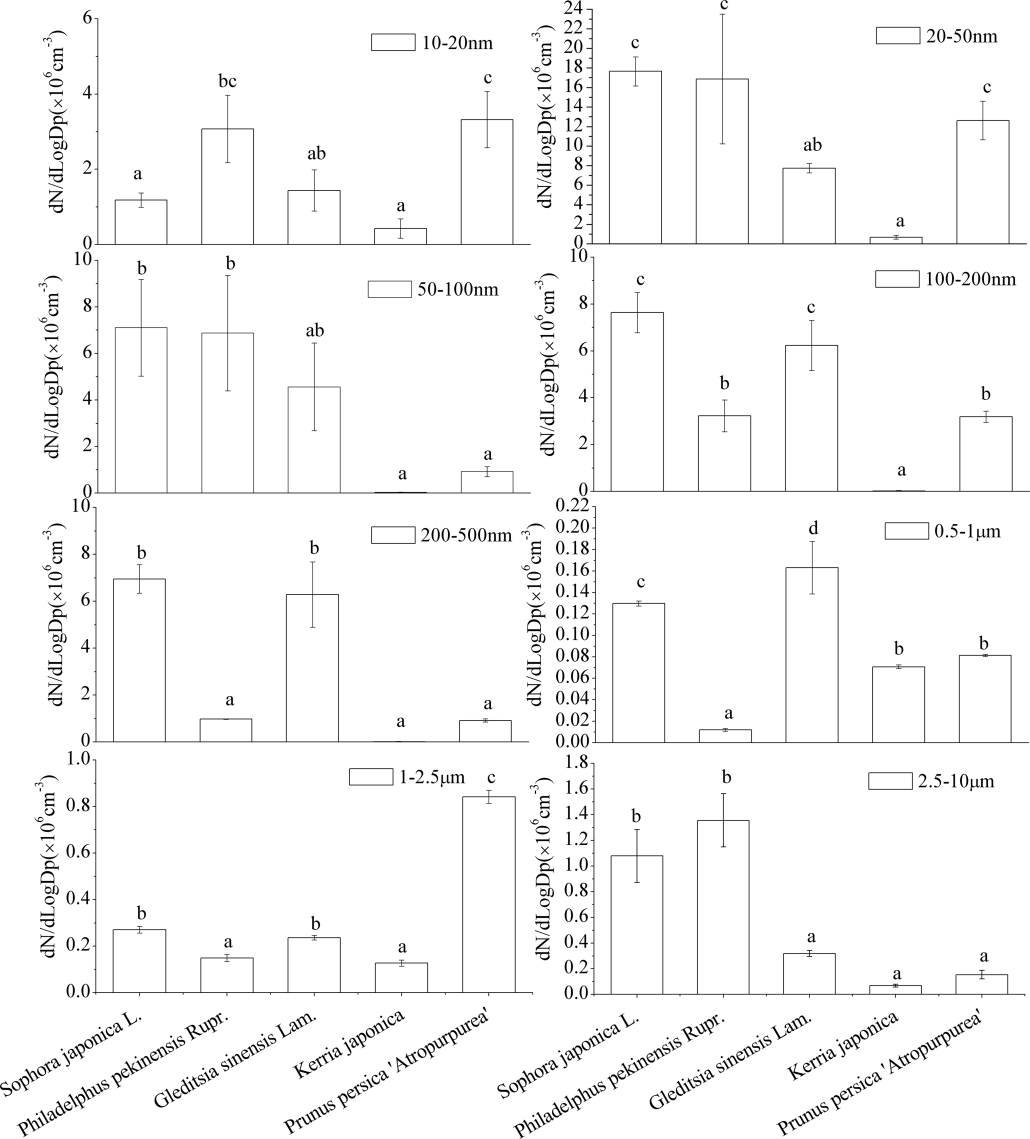
Variation in the difference between upstream and downstream measurements in particle number concentrations across all particle size classes.

Fig 2 summarizes the variation in the number concentration of all particle size classes upstream and downstream of the chamber. Deposition in the chamber increases as the upstream to downstream difference in particle number concentration increases. There were significant differences (*P* < 0.05) in particle number variation across all particle size classes among the tested tree species. For the 10–20-nm particles, the chambers containing *Prunus persica* '*Atropurpurea*' and *Philadelphus pekinensis* showed the highest particle deposition. The upstream to downstream difference of 20–50-nm particles in chambers containing *S*. *japonica*, *Philadelphus pekinensis*, and *Prunus persica* '*Atropurpurea*' were significantly higher than the differences in chambers containing *G*. *sinensis* and *K*. *japonica*. This indicates that the former species more strongly captured particles.

There were similar trends in particle number concentration in deposition chambers for particles in the 50–100-nm, 100–200-nm and 200–500-nm size ranges. *S*. *japonica*, and *G*. *sinensis* exhibited the highest deposition efficiencies, and thus there were higher number concentrations of particles in the 50–500-nm diameter size class within the plant chamber.

Considerable differences were observed in the 0.5–1-μm diameter size class among the plant species. The particle number concentrations in the *Philadelphus pekinensis* chamber were the lowest, while the concentrations in the *G*. *sinensis* chamber were the highest. The particle number concentrations in the 1–2.5-μm size class exhibited a statistically significant difference among the five test plant species; the concentration of particles in the *Prunus persica* '*Atropurpurea*' chamber samples were significantly higher than those in the others. *S*. *japonica* and *Philadelphus pekinensis* showed a tendency toward deposition of larger particles (i.e., diameters larger than 10-μm) occurring more easily and quickly, while the other three species chambers exhibited no obvious difference among PM size classes.

**Fig 3:**
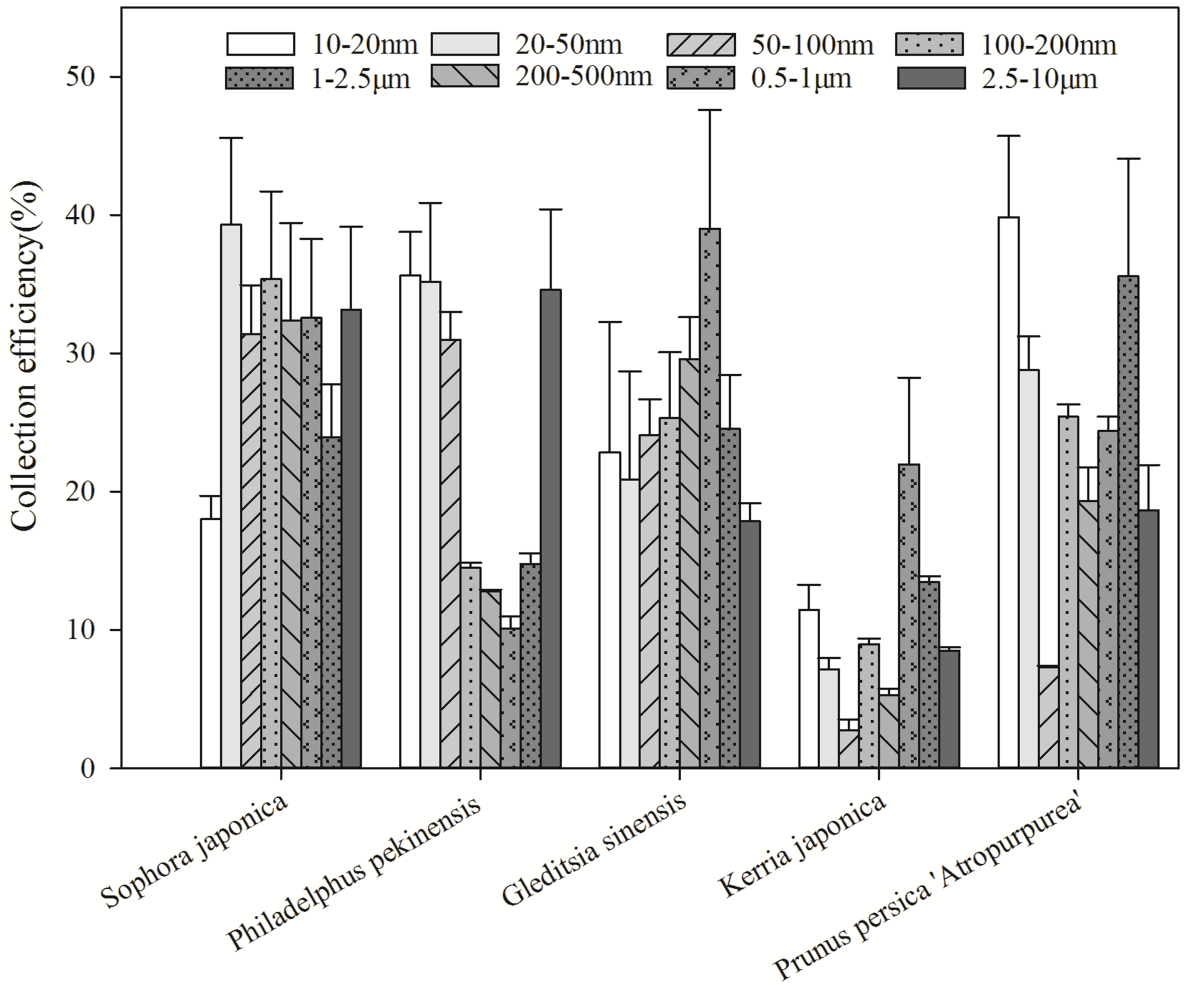
Variation in collection efficiency of each of the different particle size classes.

Fig 3 summarizes the variation in collection efficiency (*χ*_e_) in the deposition chamber across differently sized particle classes among the five tested species. The test plant chamber showed a significant reduction in PM, though the collection efficiency of all the tested plants over the whole particle size range was lower than 40%. The loss of ultrafine PM (10–20 nm) in the *Prunus persica* '*Atropurpurea*' and *Philadelphus pekinensis* deposition chamber was 39.86% and 35.64%, respectively. *S*. *japonica*, *Philadelphus pekinensis*, and *Prunus persica* '*Atropurpurea*' collected particles in the 20–50-nm size class more than other species did. The collection efficiency of particles in the 50–100-nm and 100–200-nm diameter size classes were higher for *S*. *japonica* and *Philadelphus pekinensis*, at 31.39% and 31.35%, respectively. There was a reduction of 12.81–32.36% in the 200–500-nm particle number concentration and 10.08–39.02% in the 0.5–1-μm particle number concentration in the plant simulation chamber. For the largest particle range, i.e., 2.5–10-μm, the particle number concentration throughout the monitoring periods with plants showed average reductions of 33.12% and 34.59% with *G*. *sinensis* and *S*. *japonica* in the chamber, respectively.

**Fig 4:**
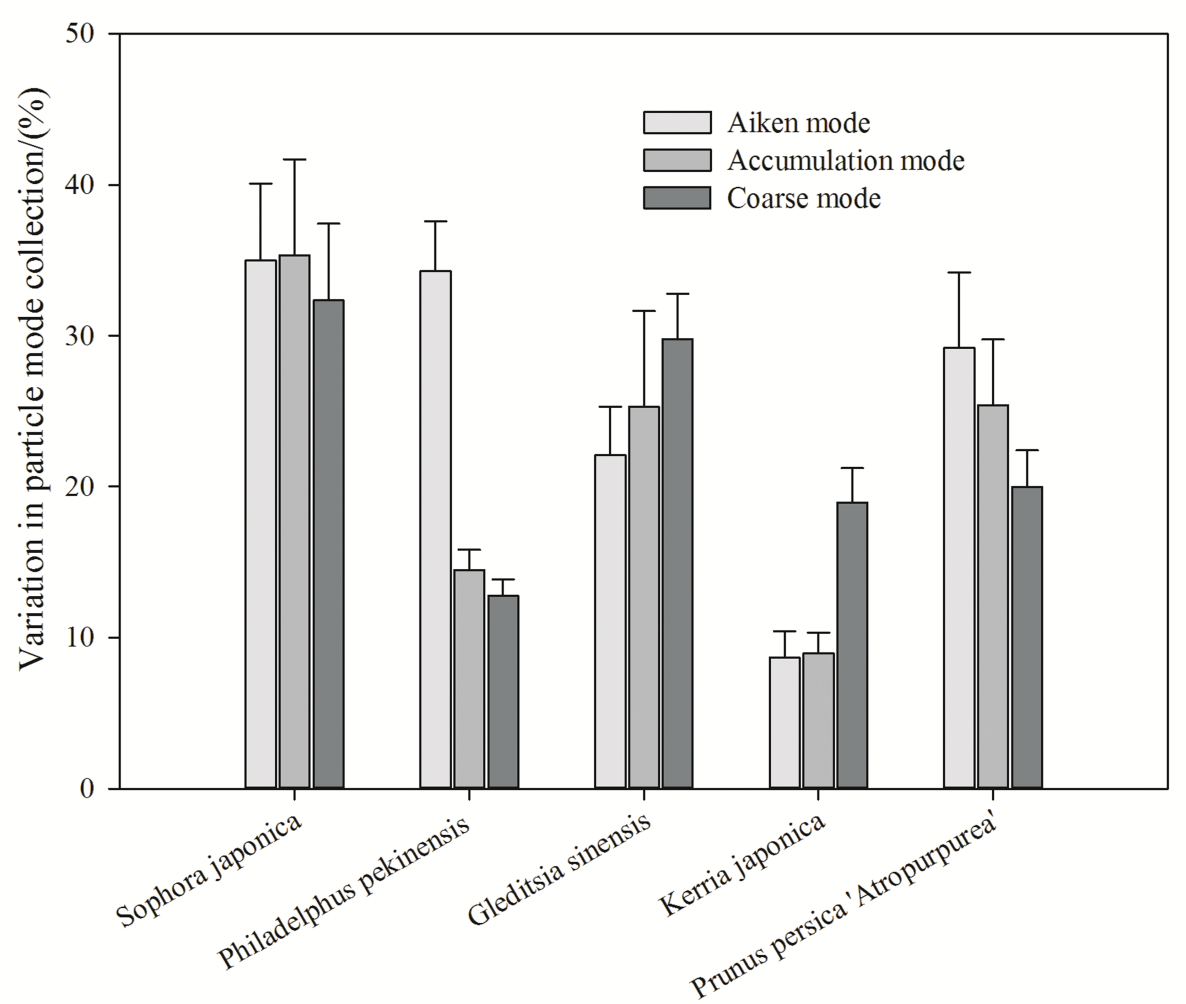
Variation in collection efficiency of each of the different particle modes captured by the five species.

Fig 4 shows the variation in particle capture among the different particle modes. The highest average number concentration percentage across the five plant species collected was that of nuclei or Aitken mode particles, reaching up to 25.85%. The average percentage reduction in the number concentration for Aitken mode particles was about 21.89%. *Philadelphus pekinensis* and *K*. *japonica* collected a substantially lower percentage of accumulation mode particles, at 12.78% and 19.99%, respectively. *S*. *japonica* exhibited a higher accumulation of coarse mode particles, at 32.26%, than the other plant species.

**Fig 5:**
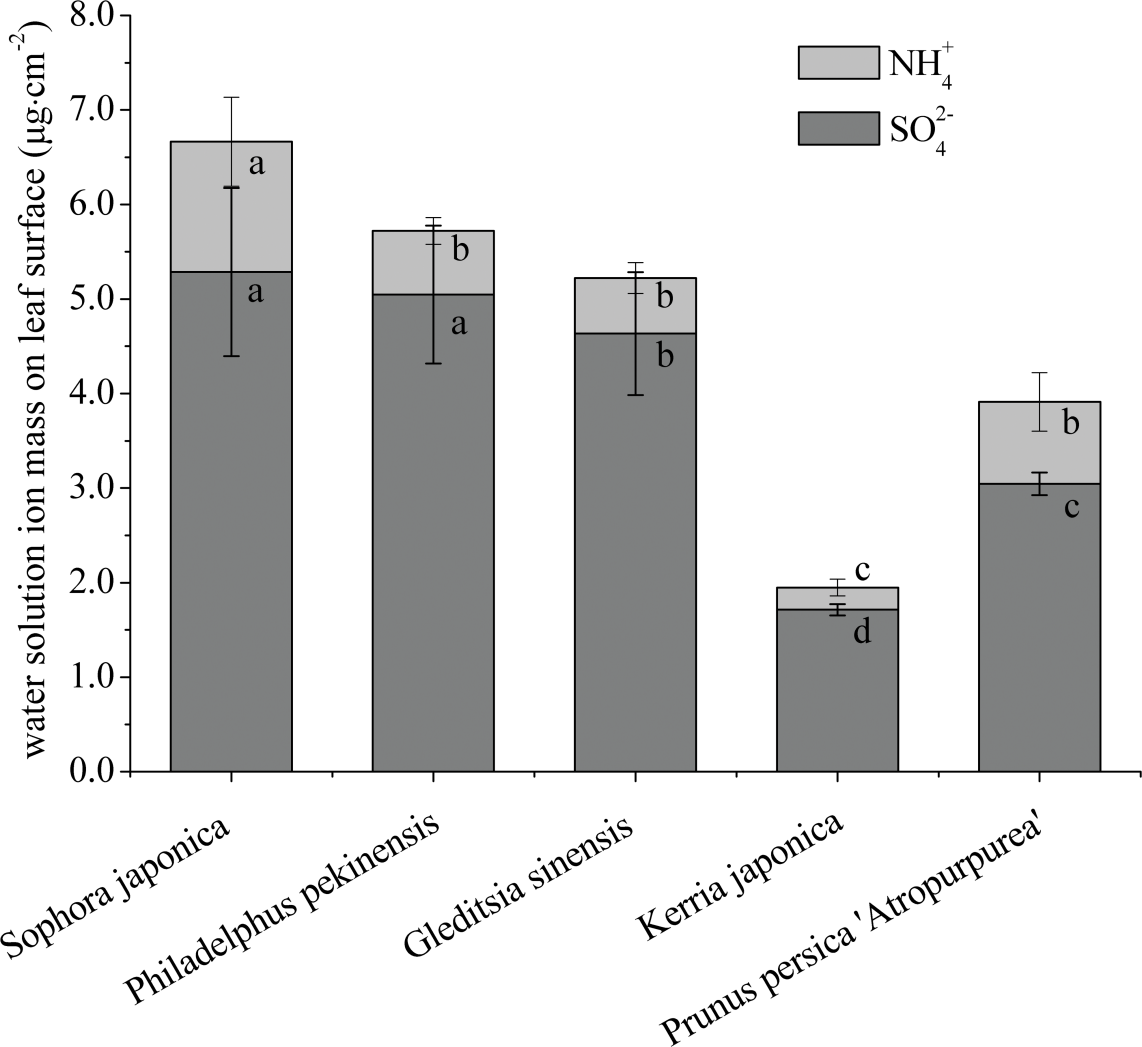
The amounts of water-soluble inorganic ions deposited on leaf surfaces.

In order to further compare the capability of tree species to capture particles, we analyzed the total mass of captured water-soluble inorganic ions in PM washed from leaf samples of each sapling. Plant species differed in their retention of surface PM on foliage (Fig 5). There was a significant difference among the washed leaves of the five tested trees (*P* < 0.05). *S*. *japonica* exhibited the highest amounts of total SO_4_^2-^ (5.286 μg·cm^-2^) and NH_4_^+^ (1.379 μg·cm^-2^) deposition on its leaf surfaces, and *K*. *japonica* had considerably lower total deposition of SO_4_^2-^ and NH_4_^+^ (1.714 μg·cm^-2^ and 0.234 μg·cm^-2^). The three remaining species showed intermediate levels of PM accumulation.

### Variation in particle deposition velocity

**Fig 6:**
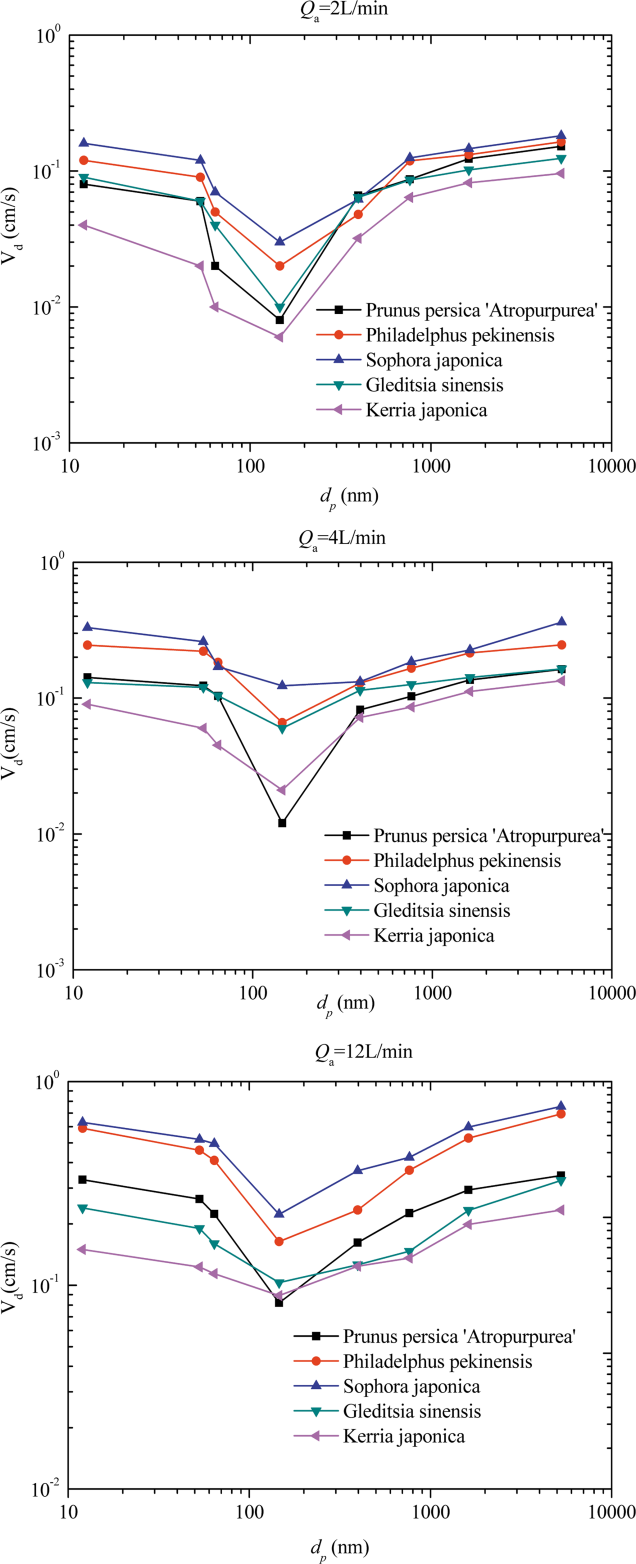
**Effects of different aerosol flowrates on the deposition velocity of each of the different tree species in the deposition chamber:** (a) 2 L·min^-1^; (b) 4 L·min^-1^; and (c) 12 L·min^-1^.

**Fig 7:**
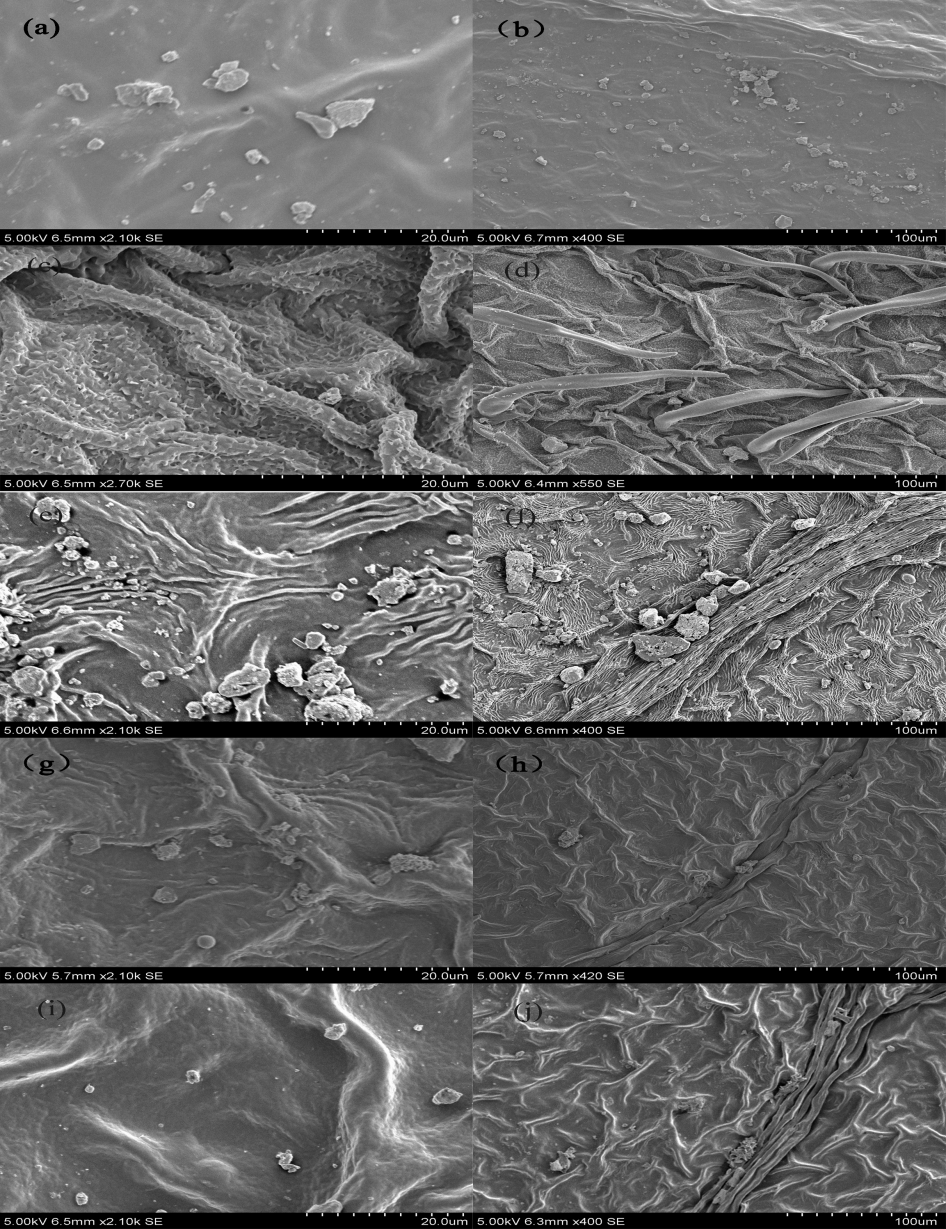
**Scanning electron micrograph of particulate matter on leaves:** (a) and (b) *Kerria japonica*; (c) and (d) *Sophora japonica*; (e) and (f) *Philadelphus pekinensis*; (g) and (h) *Gleditsia sinensis*; (i) and (j) *Prunus persica* '*Atropurpurea*.' (a), (c), (g), (e), and (i) show particles at a 20-μm scale, while (b), (d), (f), (h), and (j) show particles at a 100-μm scale.

Fig 6A–6C compare the deposition velocity at a fixed aerosol flowrate among the five tree species. Each of the deposition velocity curves were V-shaped. When the particle diameters were smaller than 146 nm, deposition velocity decreased as *d*_p_ increased owing to decreased particle diffusivity. When *d*_p_ was larger than 146 nm, deposition velocity increased with *d*_p_. For each tree species, deposition velocity also changed with the aerosol flowrate. The deposition velocity at *Q*_a_ = 12 L·min^-1^ was higher than those at *Q*_a_ = 4 L·min^-1^ and *Q*_a_ = 2 L·min^-1^. For all flowrates (i.e., 2, 4, and 12 L·min^-1^), *S*. *japonica* and *Philadelphus pekinensis* showed the highest deposition velocities, namely the greatest ability to remove airborne particles. Interestingly, among the five broadleaf trees, deposition velocity onto rough leaves was higher than that onto smooth leaves. In an effort to further explain the differences in deposition velocity of all five trees, we examined leaf surfaces by using microscopy to examine species-specific leaf traits. The leaves of *S*. *japonica* (Fig 7C and 7D) are characterized by considerable grooves and hairs on both the lower and upper sides; deep grooves can intercept more particles, decreasing the probability that PM will be released from the leaf surface. In contrast, shallow grooves make the leaf surface less rough, thereby capturing comparatively smaller amounts of particles. Additionally, plants with trichomes appeared to exhibit a positive relationship between leaf hair density and particle capture. *K*. *japonica* (Fig 7A and 7B) differed the most from *S*. *japonica*, exhibiting scarcely any tomentose pubescence and shallow grooves, consistent with the lowest measured *V*_d_ of the species.

### Variation in particle capture by plants from ambient air

The controlled experiments with artificial PM were only conducted within a defined set of fluid mechanics parameters. Accordingly, the conclusions from the controlled experiments may not necessarily be extrapolated to environments outside of this parameter space, such as ambient air.

**Fig 8:**
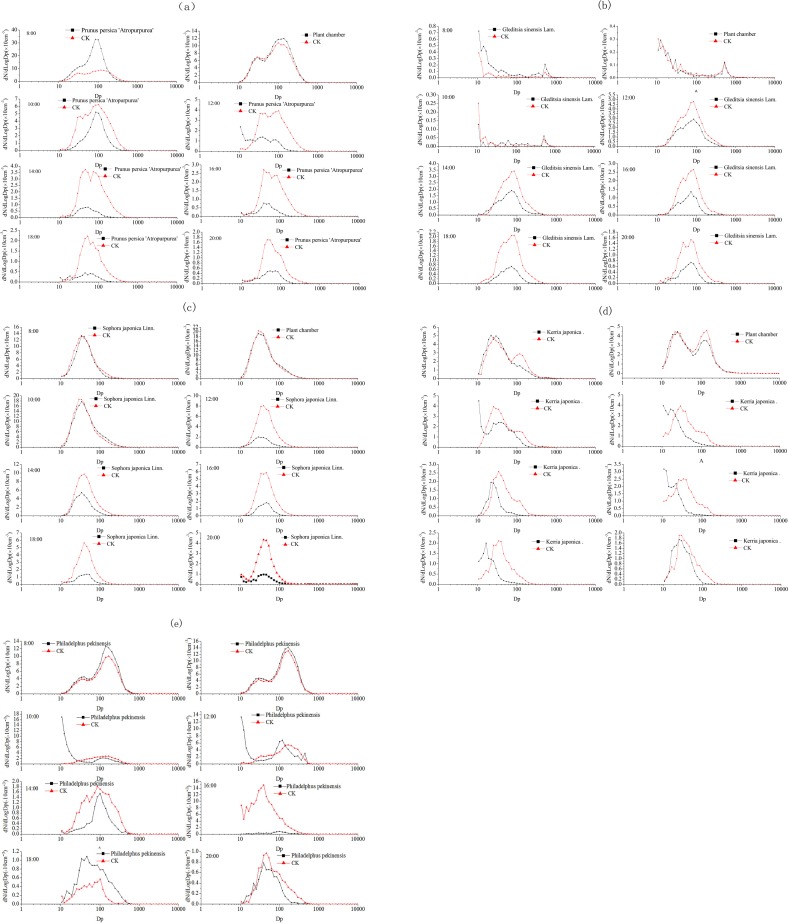
**Diurnal variation in number size distribution of particles captured by tree species in the simulation chamber:** (a) *Prunus persica* '*Atropurpurea*.';(b) *Gleditsia sinensis*; (c) *Sophora japonica*; (d) *Kerria japonica*; and (e) *Philadelphus pekinensis*

Fig 8 summarizes the average particle size distributions for *G*. *sinensis*, *S*. *japonica*, *K*. *japonica*, *Philadelphus pekinensis*, and *Prunus persica* '*Atropurpurea*' at 08:00, 10:00, 12:00, 14:00, 16:00, 18:00, and 20:00. As shown in Fig 3 and Fig 8B–8D, the particle size distributions were unimodal for *G*. *sinensis*, *S*. *japonica*, *K*. *japonica* chamber, with a distinct peak at approximately 85.8 nm at 08:00 and 10:00, 53.8 nm at 14:00 and 20:00, and 39.8 nm at 16:00. Over the course of the whole day, the particle size of the peak particle number concentrations in the simulation chambers containing plants decreased, indicating that trees do filter out PM. However, the figures also reveal that trees do not always lower the particle number concentration. For example, the particle number concentration with *G*. *sinensis* in the chamber is higher than that with the blank chamber at 8:00 (Fig 8B); similarly, the difference in particle concentration between the chamber containing *S*. *japonica* and the blank chamber was not obvious at 8:00 and 10:00 (Fig 5), while the particle number concentration within the chamber containing *Kerria japonica* was significantly higher than the blank chamber at 18:00 (Fig 8D). These findings indicated that the aerodynamic effect of the plants in the system, rather than their filtering capacities, affects the concentration variation of particles.

## Discussion

This study determined *V*_d_ values for all tested tree species across a range of flowrates. These estimates may be compared to our data from previous work, because *V*_d_ indicates the absolute effectiveness of particle capture. Some studies have confirmed that the deposition velocity of tree species differ with shoot structures. Several other research groups have examined deposition velocity (*V*_d_) and particle capture efficiency (*C*_p_) by measuring the relative deposition velocities and trapping or capture efficiencies using wind tunnels [17, 19, 23, 24].

In this study, we measured the PM capture capacities of each tree species by examining deposition velocity. *V*_d_ values changed with the aerosol flowrate; for each tree species, higher flowrates achieved higher deposition velocities, confirming the trend of enhanced particle deposition occurring at higher wind speeds, as reported by Ould-Dada et al. [16], Freer-Smith et al. [17], and Beckett et al. [24]. These researchers all agreed that *V*_d_ increases markedly with wind speed. Peter and Eiden [25] used a mathematical model to calculate *V*_d_ for particles across a range of diameters, finding *V*_d_ values of 0.02 cm·s^-1^ at wind speeds of 0.5 m·s^-1^ for 1-μm particles. Freer-Smith et al. [17] measured *C*_p_ and *V*_d_ values using similar empirical techniques and wind speeds for three European broadleaved tree species and two conifers; the deposition velocities ranged from 0.1 to 0.3 cm·s^-1^ at a wind speed of 3 m·s^-1^ to a maximum value of 2.9 cm s^-1^ at a 9-m·s^-1^ wind speed. Both the *V*_d_ and *C*_p_ values were greater for the needles of conifers than the leaves of broadleaf species. However, the influence of wind speed on *V*_d_ and *C*_p_ differed. Wind speed had a greater influence on *V*_d_ for both leaves and stems than *C*_p_; this was because *C*_p_ values are relatively small for stems and show a weaker relationship with wind speed. Beckett et al. [24] conducted wind tunnel experiments with NaCl particles, revealing a clear relationship between wind speed and deposition velocity, consistent with greater wind speed imparting larger particles with more inertia and, hence, more effective impaction. Lin and Khlystov [19] conducted a study that focused on ultrafine particle deposition onto vegetative branches; ultrafine particle removal efficiency was found to increase with particle size and wind speed, but decrease with packing density. Each of these studies were based on the relationship of wind speed with *V*_g_ and *C*_p_. The theoretical expectation was that a number of particles directly strike an object rather than being diverted around it. Therefore, the momentum of the particle increases with wind speed, which facilitates particles penetrating the boundary layer more effectively and thereby increases the retention probability of impacted particles.

However, higher wind speeds did not always increase particle deposition. Higher wind speeds can lead to particles bouncing off or becoming resuspended. In field experiments, McPherson et al. [26] found that 50% of particles captured by trees may be resuspended. However, in the wind tunnel simulation conducted by Beckett et al. [24], particles did not bounce off at wind speeds of up to 9 m·s^-1^.

Another important factor with great influence on *V*_d_ and *C*_p_ was particle size. Our data showed that *V*_d_ values were higher for ultrafine particles and decreased with size, reaching a minimum at *d*_p_ = 146 μm. The mechanism of particle movement differs with particle size. For particles with a diameter of *d*_p_ > 10 μm, sedimentation is the key deposition process. For particles with a diameter of *d*_p_ < 10 μm, both deposition and acceleration caused by gravity were generally decreased. Between 0.1-μm and 1-μm diameters, impaction and interception are the main processes acting on particles in the air. Only diffusion is effective for particles with a diameter of *d*_p_ < 0.1 μm, but this still results in high deposition rates.

Above all, however, turbulent air flow and associated impaction are the main mechanisms resulting in greater deposition on trees than on shorter vegetation. The inertia of particles travelling in an air stream as it curves around an object, such as a leaf or stem, forces them through the boundary layer and onto the object’s surface [27]. The deposition rate for submicron particles was 10–30 times higher on grass than a cement surface [28]. Ultrafine particles diffuse in random directions owing to Brownian motion, and the deposition velocity can be represented by the mean mass diffuse transfer coefficient, which is correlated with the Reynolds number, as reported by some studies [29–31]. When flowrates increase, the flow velocity in the deposition chamber increases, i.e., the Reynolds number becomes larger, resulting in a higher mean mass transfer coefficient. In other words, for ultrafine particles, the deposition velocity increases with flowrate, as has been shown by many studies [30, 32].

In the present study, when the particle diameters exceeded 146 nm, inertial deposition and gravitational settling played an important role, and the *V*_d_ of particles in this size class increased with size owing to increased impaction rates. However, some research has also reported that *V*_d_ for submicron particles decreased with size [19, 33, 34]. Deposition velocities for 0.01–0.1-μm diameter particles at wind speeds of 0.3–1.5 m·s^-1^ were assessed, and *Cupressus leylandii* and *Pinus sylvestris* hedges were found to be effective filters of particles within this diameter range, like pine needles [35]. These studies confirmed earlier findings that deposition velocity decreased with size for submicron particles. In our study, more ultrafine particles were captured across the five different plant species, indicating that ultrafine particles are more easily deposited on leaf surfaces and that the deposition velocities are large. Few studies have examined the number of particles on leaf surfaces of different plant species. However, available empirical estimates are of the same order of magnitude as those in this study. For example, Freer-Smith et al. [17] found that deposition velocities were higher for ultra-fine particles than for coarse and fine particles. Terzaghi et al. [36] observed that particle sizes deposited on leaf or needle surfaces ranged from 0.2 to 70.4 μm; fine particles dominated the distribution, representing between 70% (for *Cornus mas*) and 88% (for *Acer pseudoplatanus*) of the total particle count. Ottelé et al. [37] and Teperet al. [38] presented similar findings, concluding that particles with diameters larger than 10 μm were scarce compared to particles with diameters <10 μm and that particles (with diameters less than 2.5 μm) dominated leaf or needle surfaces of different plant species. Indeed, the different size-fractioned particles in the simulation chamber may have been transformed into different modes. Submicron particles contribute substantially to particle number concentrations [39], and new particle formation, associated with a rapid burst of nucleation mode particles, increases the total number concentration of submicron particles [40]. The particle formation rates are significantly higher in different geographic regions and under different meteorological conditions; field observations have revealed that formation rates can reach up to 0.01–10 cm^-3^·s^-1^ in the boundary layer and higher in urban areas and 10^4^–10^5^ cm^-3^·s^-1^ in coastal areas [41].

The properties of plant species and leaf surfaces have considerable impacts on deposition velocity. Rough surfaces increase deposition compared to smooth surfaces, and most plants have a high surface area per unit volume. Wind tunnel tests [24] have shown that capture efficiency and deposition velocity of NaCl particles is significantly higher on certain conifers (e.g., pine and cypress) than deciduous trees (e.g., maple and poplar) as a result of the more complex spatial structure of the former. The foliage structures of conifers are distributed into fine cylinders, while those of broadleaf species represent broad, flat planes; because airflow is less turbulent across a large plane than a fine cylinder, there are dramatic differences between conifers and deciduous trees in their aerodynamic characteristics.

It should also be noted that the differences in deposition velocity among all tested plant species were impacted by leaf epidermis structure, i.e., the surface characteristics of tree leaves such as hairs, grooves, veins, and other structures on the leaves shown in Fig 8A–8J. It has been suggested that hairy and sticky leaves may capture more particles. The leaf surfaces of *S*. *japonica* have been described as having pubescence and substantially more pronounced grooves (Fig 7C and 7D), which induced the highest *V*_d_ values. *Philadelphus pekinensis* was similar to *S*. *japonica* with respect to its substantial grooves, but it lacked trichomes (Fig 7E and 7F); similarly, it had a higher *V*_d_ value. *G*. *sinensis* was the most unlike *S*. *japonica*, having more wax and thicker leaves (Fig 7G and 7H). *K*. *japonica* had the smoothest leaf surfaces (Fig 7A and 7B). *Prunus persica* '*Atropurpurea*' has been described as having grooves but relatively low coverage of hairs (Fig 7I and 7J). It should be noted that the unit leaf area of *S*. *japonica* exceeded that of *K*. *japonica*, and its mean *V*_d_ value was 1.5–2 times higher.

However, leaf area was not proportional to particle capture for all species. Räsänen et al. [20] demonstrated that smaller conifer needles increase particle capture efficiency. For broadleaved trees, this was not reflected in *C*_p_, though pubescent birch and silver birch had leaves that were two times smaller than those of lime. In the case of the broadleaf trees, therefore, leaf traits (i.e., roughness, trichomes, grooves, and other structures) seemed to play an important role in removing aerosol particles. Physiological characteristics of leaves, such as stomatal density, transpiration, stomatal conductance, and wettability, also have significant influence on particle capture efficiency. In general, *C*_p_ was increased under low stomatal density, high stomatal conductance, high transpiration, and low wettability for broadleaved species [20]. In addition to the above differences among species, deposition velocity and capture efficiency also differ among parts of the same tree species, e.g., leaves and stems [17].

In addition to their impact on airstream turbulence and particle capture, plants may also be sources of particle emission under certain conditions. For example, volatile organic compounds (VOCs) emitted by plants are an important factor that can highly increase the abundance of secondary organic aerosol (SOA), which is an important gas precursor in the formation of atmospheric particles. Plant species have specialized structures and organs that store and emit VOCs (e.g., glandular trichomes and resin ducts) [42]. The main VOC components from plants consist of isoprene, monoterpenes (such as α/β-pinene), and sesquiterpenes (such as β-caryophyllene), all of which are oxidized by ozone and •OH radicals, thereby generating SOAs [43].

In our simulation experiment, the amounts and quality of accumulated PM by plants was affected by biogenic VOCs. Only a few studies have examined the oxidation of VOCs and their role in SOA formation under laboratory simulation conditions [44, 45]. In order to better understand the effects of atmospheric ozone levels on plant monoterpene emissions and on SOA formation, Pinto et al. [46] conducted an experiment in which monoterpenes were mixed with an air-flow enriched with 100, 200, or 400 ppb by volume of ozone in a Teflon-coated simulation chamber. No new particles were formed, but clear SOA formation was observed at higher ozone concentrations. The most common types of oxides are ozone and •OH radicals, so it is also important to compare the relative importance of OH oxidation with ozonolysis of monoterpenes in particle nucleation and growth. Some studies have shown the importance of ozonolysis in new particle formation [47, 48], while others have emphasized the importance of OH oxidation [49, 50]. However, some studies have found that growth rates for each particle size class did not necessarily correlate with the reaction rate of monoterpenes with OH and ozone [51]. In future work, these above factors should be considered in order to determine the factors and compounds that contribute to particle growth; this will be the next focus of our research.

Although the previous deposition chamber trial found that plants in such systems can absorb particle pollutants through their stomata, particles are also removed from the system by deposition onto the leaves and branches at different deposition velocities that differ among tree species. However, we also conducted these experiments under considerably different environments, i.e., a plant chamber (and blank chamber) through which ambient air was pumped, and we still found these trees make a difference. Some of the test trees did not continually reduce the PM concentration in the system; this pattern is consistent with plants in the chamber decelerating the flows in and out of the system, causing the particles to become highly concentrated in the system as there is less air flow available for dilution. This result therefore casts doubt on whether trees can improve air quality. There are some studies that predict urban forests can remove pollutants at the city scale [10, 52, 53]. This previous research is based on a deposition model [54] with limited air quality improvement effects of forests (i.e., 1–2%) [10]; however, the lack of empirical evidence for these deposition models supports the simulation finding bolstered by the current study that the air quality improvement of urban forests is overestimated [55].

In this study, though particles from both the manipulated aerosol and ambient air were deposited on to the leaf surfaces resulting in decreased concentrations at some point, but under certain circumstances PM concentration actually increased. Similar studies [56, 57, 58] have demonstrated that trees along urban streets obstruct wind flow thereby reducing the exchange of air and leading to higher pollutant concentrations at ground level. Vos et al. [59] found that roadside urban vegetation actually leads to increased pollutant concentrations rather than improves air quality. Accordingly, we cannot precisely determine which tree species are optimal for capturing dust and thus mitigating air pollution. In short, the negative effect of vegetation on the local air quality should not be neglected, especially by policy makers.

The initial goal of this study was to determine which tree species best captures and removes particles and can therefore be used in afforestation to improve local air quality. This research indicates that it is difficult to make recommendations, even though trees do absorb or capture air pollutants in a way that is predictably affected by the structure of their organs. Complex branching patterns and leaf structures also change and limit air flow that dilutes pollution at ground level, thereby leading to the significantly higher PM concentrations. This aerodynamic effect seems to outweigh the filtering capability of vegetation under some conditions. As such, we still need some empirical evidence and field survey data to confirm the results of this and similar previous research.

These results and conclusions must be considered in a broader context. While some trees did not effectively reduce airborne PM in our research limited to indoor simulation chamber conditions, this does not necessarily mean that trees in urban backyards, in parks, and along streets have a similar effect. In addition, urban forests both have an important role as structures in urban environments and provide social and ecosystem services to metropolitan areas. Overall, there are conflicting effects of trees in urban spaces with respect to reducing PM pollution. This poses a question that must be considered; which is more important in reducing the PM concentration, the physical characteristics of vegetation or the air flow impacts of trees in urban environments? If the physical characteristics of vegetation are more important, tree species with complex leaf structures and a maximum of foliage should be selected. However, if impacts on air flow are more important, urban spatial planting configurations should be selected that optimize the dilution of pollution by assessing air flow at a micrometeorological scale.

## Conclusions

Five typical tree species common to north China were selected to investigate PM removal in a deposition chamber. Deposition velocity onto trees was calculated for each tree species in order to quantitatively compare the capability of tree leaves to remove particles of different sizes. The deposition velocity onto S. *japonica* was highest. Thus, *S*. *japonica* showed the greatest capability to remove particles, followed by *Philadelphus pekinensis*, *G*. *sinensis*, *Prunus persica* '*Atropurpurea*', and *K*. *japonica*, in descending order. In broadleaf trees, the ability to remove particles was strongly affected by leaf structures, i.e., rough leaf surfaces more easily removed particles. However, under a different indoor simulation condition using ambient air, this observed capability for capturing PM was not obvious. Species differences in removing PM are clear, but these results do not necessarily predict the effects of these species in practical application owing to the effects of specific habitat conditions. Accordingly, policy makers and the public should reconsider their perspectives on local pollution problems; intuitively, trees are sought as a means to alleviate air pollution problems, the counterintuitive negative effects of vegetation on local air quality must also be considered. Hence, when selecting tree species to efficiently reduce particle air pollution and improve air quality in urban environments, a key question must be asked. How can trees be used without significantly deteriorating the local air quality? In answering this question, the physical characteristics of individual tree leaf structures must be considered as well as appropriate designs for the configuration of planting patterns in order to facilitate airflow to improve local air quality.

## Supporting information

S1 File Data used to analyze variation in average particle concentrations and PM captured by different tree species. There are two related datasets in the XLSX file: (1) concentrations of different particle sizes across the five tree species used in the simulation chamber and (2) accumulations of the different sized particles on leaf surfaces and in the wax layer.
(XLSX)
